# Value-based healthcare in ischemic stroke care: case-mix adjustment models for clinical and patient-reported outcomes

**DOI:** 10.1186/s12874-019-0864-z

**Published:** 2019-12-05

**Authors:** Arvind Oemrawsingh, Nikki van Leeuwen, Esmee Venema, Martien Limburg, Frank-Erik de Leeuw, Markus P. Wijffels, Aafke J. de Groot, Pieter H. E. Hilkens, Jan A. Hazelzet, Diederik W. J. Dippel, Carla H. Bakker, Helene R. Voogdt-Pruis, Hester F. Lingsma

**Affiliations:** 1 Center for Medical Decision Making, Department of Public Health, Erasmus University Medical Center, PO Box 2040, 3000 CA Rotterdam, the Netherlands; 2 Department of Neurology, Erasmus University Medical Center, Rotterdam, the Netherlands; 3 Department of Neurology, Flevoziekenhuis, Almere, the Netherlands; 4 Stroke Knowledge Network Netherlands, Utrecht, the Netherlands; 5 Department of Neurology, Donders Institute for Brain Cognition and Behaviour, Radboud University Medical Center, Nijmegen, the Netherlands; 6 Department of Neurorehabilitation, Rijndam Rehabilitation, Rotterdam, the Netherlands; 7 Department of General Practice and Elderly Care Medicine, Amsterdam University Medical Center, Amsterdam, the Netherlands / Vivium Naarderheem, Naarden, the Netherlands; 8 Department of Neurology, St. Antonius Hospital, Nieuwegein, the Netherlands; 9 Expert Centre Quality Registries, Leiden University Medical Center, Leiden, the Netherlands; 10 EnCorps, Hilversum, the Netherlands

**Keywords:** Ischemic stroke, Case-mix, Risk adjustment model, Patient-reported outcome measure, Value-based healthcare

## Abstract

**Background:**

Patient-Reported Outcome Measures (PROMs) have been proposed for benchmarking health care quality across hospitals, which requires extensive case-mix adjustment. The current study’s aim was to develop and compare case-mix models for mortality, a functional outcome, and a patient-reported outcome measure (PROM) in ischemic stroke care.

**Methods:**

Data from ischemic stroke patients, admitted to four stroke centers in the Netherlands between 2014 and 2016 with available outcome information (*N* = 1022), was analyzed. Case-mix adjustment models were developed for mortality, modified Rankin Scale (mRS) scores and EQ-5D index scores with respectively binary logistic, proportional odds and linear regression models with stepwise backward selection. Predictive ability of these models was determined with R-squared (R^2^) and area-under-the-receiver-operating-characteristic-curve (AUC) statistics.

**Results:**

Age, NIHSS score on admission, and heart failure were the only common predictors across all three case-mix adjustment models. Specific predictors for the EQ-5D index score were sex (β = 0.041), socio-economic status (β = − 0.019) and nationality (β = − 0.074). R^2^-values for the regression models for mortality (5 predictors), mRS score (9 predictors) and EQ-5D utility score (12 predictors), were respectively R^2^ = 0.44, R^2^ = 0.42 and R^2^ = 0.37.

**Conclusions:**

The set of case-mix adjustment variables for the EQ-5D at three months differed considerably from the set for clinical outcomes in stroke care. The case-mix adjustment variables that were specific to this PROM were sex, socio-economic status and nationality. These variables should be considered in future attempts to risk-adjust for PROMs during benchmarking of hospitals.

## Background

The growing trend to benchmark certain health care performance indicators – to assess the health care quality between institutions – requires careful consideration of the methodology that is being used [1, 2]. Healthcare is evolving towards a value-based healthcare framework with more emphasis on Patient-Reported Outcome Measures (PROMs), that will not only facilitate opportunities for performance improvement at an individual patient level when these measures are used in clinical practice, but may also be useful for benchmarking across providers [3–5]. PROMs can be defined as feedback directly from the individual patient on their own health condition (e.g. symptoms and health-related quality of life), thus without external interpretation [6]. PROMs can be either disease-specific (e.g. Neuro-QOL [7]) or generic (e.g. EQ-5D [8]).

An important consideration for meaningful comparisons across hospitals is the case-mix adjustment of the patient populations for each health care provider [9, 10]. By adjusting for the heterogeneity of patient characteristics in inter-hospital comparisons, a larger part of the estimated variation between hospital performances will be attributable to the quality of care provided to patients rather than factors outside of the healthcare providers’ control.

In stroke, the most commonly used clinical outcome measures are mortality and the modified Rankin Scale (mRS). There has been considerable research conducted on prognostic models for these clinical outcomes, which also encompass variables for case-mix adjustment [11]. Although there is a strong trend to use PROMs for benchmarking purposes [12], there still remains a lack of case-mix models to predict patient-reported outcomes as compared to clinical outcomes [11]. The aim of this study was to identify the specific variables for case-mix adjustment for a generic PROM (EQ-5D) and compare them to case-mix variables for clinical outcomes in acute ischemic stroke.

## Methods

### Patient population and data collection

A core set of baseline patient characteristics, performance indicators and outcome measures were registered from March 2014 till August 2016 of four stroke care centers in the Netherlands, of which 1 was a university and 3 were district-based hospitals. The original database contained consecutive acutely admitted ischemic stroke patients of which demographic, process indicators and outcome measures were registered.

**Fig. 1:**
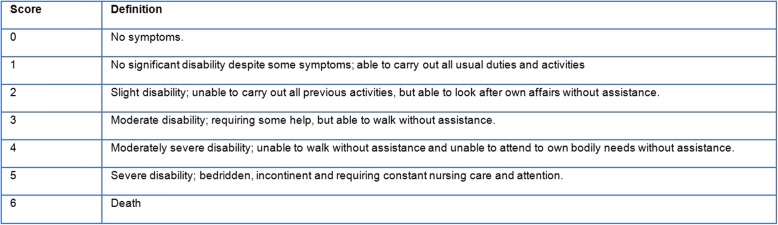
Modified Rankin Scale (mRS)

The three outcome measures were mortality at 3 months, modified Rankin Scale (mRS) score at 3 months and EuroQol-5D index score at 3 months. The mRS is a commonly used clinician-reported scale, which measures the degree of disability after a stroke, with scores ranging from 0 to 6 (Fig. 1) [13]. The mRS score at 3 months post-discharge was generally recorded by trained nurses, either by phone or at the outpatient clinic. The EQ-6D, a generic health-related quality of life (HRQOL) instrument, is based on the EQ-5D (dimensions: usual activities, self-care or autonomy, mobility, pain/discomfort, and anxiety/ depression) with an additional question on cognitive functioning. The survey has been translated into Dutch and validated in previously published literature [8, 14, 15]. The post-discharge EQ-6D data was captured through either face-to-face or telephone interviews with patients themselves or their proxies. Due to the lack of a validated EQ-6D index tariff, the utility score was derived and transformed through the EQ-5D index tariff, by ignoring the “cognitive” dimension of the EQ-6D [16–18]. This EQ-5D tariff is an algorithm for attaching values to all 3125 health states often used in economic evaluations. This utility score can be used to compare to population norms or to calculate quality-adjusted life years (QALY’s) [16, 17, 19]. The authors will, for the remainder of this article, solely mention “EQ-5D index score” as the patient-reported outcome of interest to avoid any confusion. This EQ-5D index score ranged from 0 (death) to 1 (perfect health) and signified the patient’s perspective on his/ her own health. Because there still is no consensus on the minimal clinically important difference on the EQ-5D utility score in stroke populations [20], it was decided to keep the EQ-5D utility score as a continuous outcome rather than modify it to an ordinal outcome based on arbitrary cut-offs.

**Fig. 2:**
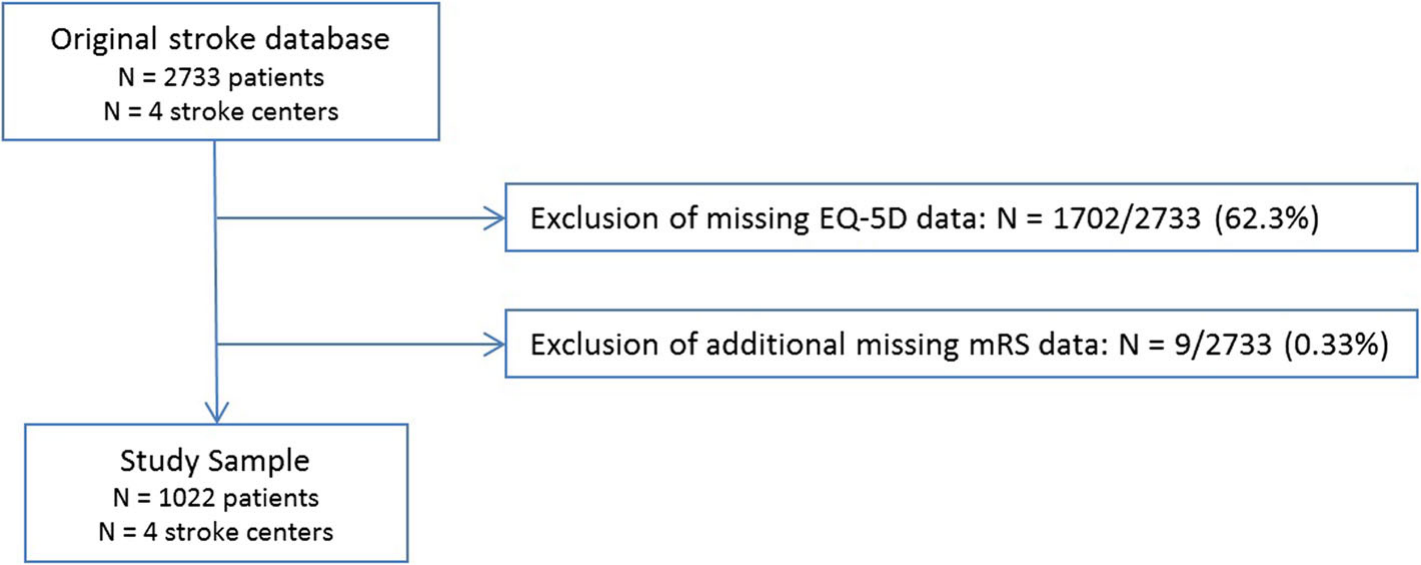
Flowchart of Study Population Selection

Missing baseline patient characteristics (among which case-mix variables) were imputed 10 times using multiple imputation in the original database (*N* = 2733 patients of 4 stroke centers), assuming missingness at random. Predictors (including stroke center) and outcomes also served as indicators for the imputation model [21]. Figure 2 showcases the substantial proportion of missing mRS and EQ-5D data that were filtered out before the three case-mix adjustment models were developed. Thus, all three regression models were developed using an imputed (10 iterations) dataset also containing the “original” 1022 patients. Potential reasons for missing patient-reported outcome data were patients being too sick to fill questionnaires out, and loss-to-follow at 3 months (patients being unreachable due to staying at a nursing home/ rehabilitation center, or because of their tremendous recovery).

**Fig. 3:**
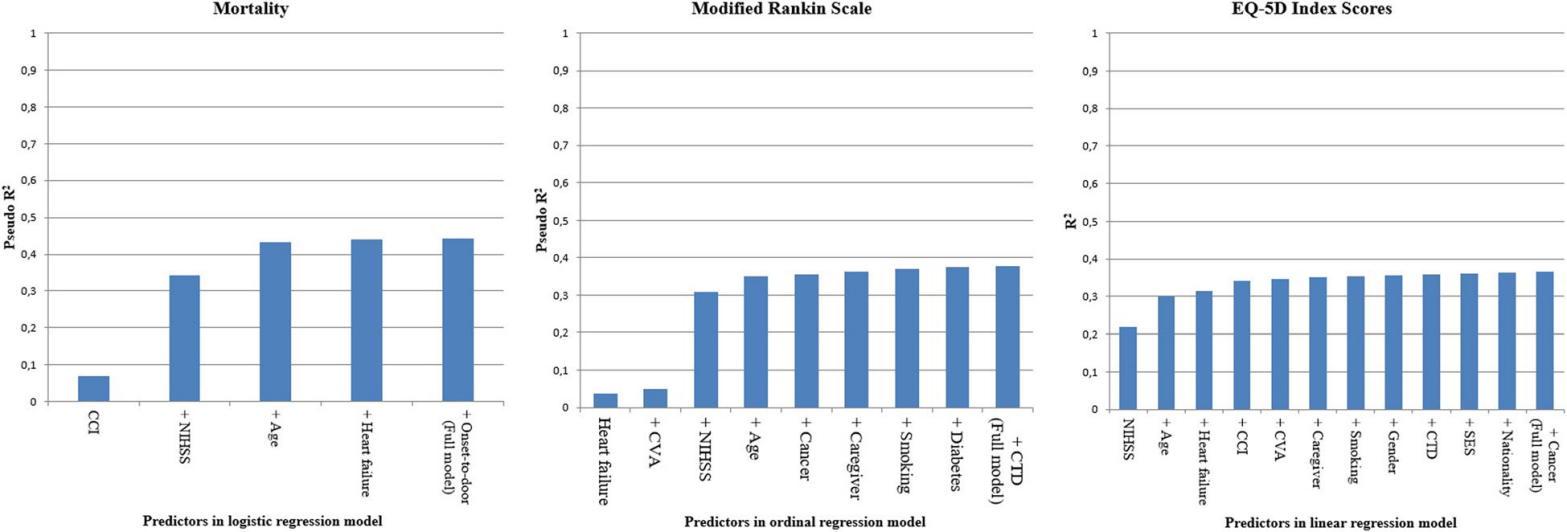
Prognostic Value of Univariable and Full Models for Three Outcomes, Expressed in Percentage Explained Variance (R^2^)

### Case-mix models

Patient characteristics that could differ between hospitals and could be predictive of outcomes were considered potential candidate case-mix variables and were identified based on clinical experience and past literature. Those included: age, sex, nationality, socio-economic status (SES), smoking, cardiovascular comorbidity (e.g. hypertension, hyperlipidemia), stroke in past history, diabetes, cancer, connective tissue disease, Charlson comorbidity index (CCI), stroke onset-door time, initial National Institutes of Health Stroke Scale (NIHSS) score and the presence of a caregiver. The SES was generated by the ranking of status scores (based on neighborhoods/ zip codes) that have been calculated and published by Social and Cultural Planning Office (SCP), a Dutch governmental institution [22, 23].

### Statistical analysis

Descriptive statistics were presented as counts (percentages) or median ± inter-quartile range (IQR). Nonparametric tests were used where appropriate to determine (unadjusted) differences in patient populations between the four healthcare providers, using the Pearson chi-squared statistic for categorical variables and the Kruskal-Wallis test for continuous variables. A *p*-value < 0.05 was considered significant. To assess the adjusted effect of the potential case-mix independent variables, the models were developed using logistic, ordinal and linear regression models respectively for mortality, mRS score and EQ-5D index score with stepwise backward selection. This stepwise (regression-based) method initially tests all the predictors in a regression model and subsequently eliminates the least significant variables in a stepwise approach with a certain cut-off *p*-value [21]. In this study, the AIC (Akaike information criterion) [24], equivalent to *p* < 0.157, was used as a criterion.

For the logistic and ordinal regression models, the odds ratios (ORs) with 95% confidence intervals (CI) were calculated per predictor. Beta’s (ß’s) and 95% CI were calculated for predictors in the linear regression model. The ß coefficient indicates the change in outcome (units on the EQ-5D index score scale) for one unit change in the predictor variable. The ability of the case-mix models to explain the variability (‘goodness-of-fit’) of these 3 outcomes was expressed by calculating the R^2^ (R-squared) statistics [25]. The predictors for each model were added in consecutive order based on the *p*-values (lowest to highest p-value) and coefficients. The explained variance of each additional predictor was demonstrated till each model was completed.

Because the R^2^ measure is not immediately comparable between different regression models (logistic vs. ordinal vs. linear), the AUC (area-under-the receiver-operating-characteristic-curve) statistic was also included to get a sense of the comparability between the three risk-adjustment models. For this additional analysis, both mRS (0–2 vs. 3–6) and EQ-5D (< 0. 65 vs. ≥ 0.65) were transformed to a binary outcome variable in order to compare the three logistic regression models. The EQ-5D index score of 0.65 was chosen as a cut-off value, as it was the estimated median score in this study sample. The statistical analysis was carried out by using IBM SPSS Statistics 21 & RStudio version 1.0.153.0-© 2009–2016 RStudio, Inc. software.

## Results

### Patient characteristics

In total, 1022 ischemic stroke patients were included. The number of patients per studied hospital varied from 29 to 555 patients. 57% of total study participants were men (Table 1). The unadjusted median age was significantly different (range 70–78 years, *p* = 0.001) across the four stroke care centers. Most patients (87%) were native Dutch inhabitants. Both the Charlson Comorbidity Index and the stroke rate in patient history were similar across the 4 stroke patient cohorts. There was a significant unadjusted difference (range 113–275 min, *p* = 0.002) in the onset-to-door time between the patient populations of the four stroke care centers.

**Table 1:** Characteristics of all Ischemic Stroke Patients (*N* = 1022) and per Dutch Stroke Center, Admitted from March 2014 – August 2016 in Four Dutch Stroke Hospitals

Patient characteristics	All patients (*N* = 1022)	Stroke Center I, University (*N* = 222)	Stroke Center II, District-based (*N* = 555)	Stroke Center III, District-based (*N* = 216)	Stroke Center IV, District-based (*N* = 29)	*P*-value	Missing data, N (%)
Male, N (%)	578 (57)	139 (63)	315 (57)	109 (50)	15 (52)	0.076	
Age, median (IQR)	74 (64–82)	70 (59–80)	76 (66–83)	72 (63–82)	78 (72–85)	0.001	
Nationality						0.351	84 (8)
Native Dutch	888 (87)	171 (77)	517 (93)	174 (81)	26 (90)		
Foreigner	50 (5)	9 (4)	34 (6)	5 (2)	2 (7)		
Smoking, N (%)	225 (22)	51 (23)	131 (24)	37 (17)	6 (21)	0.604	72 (7)
SES, N (%)						< 0.001	8 (1)
Low	335 (33)	101 (45)	131 (24)	81 (38)	22 (76)		
Middle	427 (42)	94 (42)	214 (39)	119 (55)	0 (0)		
High	252 (25)	25 (11)	207 (37)	15 (7)	5 (17)		
NIHSS on admission, median (IQR)	4 (2–12)	5 (2–9)	4 (2–14)	3 (1–8)	3 (2–14)	0.028	75 (7)
CCI, median (IQR)	1 (0–2)	1 (0–2)	1 (0–2)	1 (0–2)	1 (0–3)	0.133	89 (9)
Comorbidities, N (%)							
Hypertension	546 (53)	123 (55)	332 (60)	74 (34)	17 (59)	< 0.001	9 (1)
Myocardial infarction	103 (10)	15 (7)	71 (13)	17 (8)	0 (0)	0.012	15 (2)
Heart failure	66 (7)	2 (1)	52 (9)	8 (4)	4 (15)	< 0.001	22 (2)
Previous stroke/TIA	274 (27)	58 (26)	156 (28)	54 (25)	6 (21)	0.745	10 (1)
Carotid stenosis	64 (6)	11 (5)	44 (8)	8 (4)	1 (3)	0.093	50 (5)
PAOD	80 (8)	13 (6)	46 (8)	17 (8)	4 (14)	0.374	14 (1)
Diabetes mellitus	39 (4)	9 (4)	25 (5)	4 (2)	1 (3)	0.409	15 (2)
Connective tissue disease	26 (3)	0 (0)	13 (2)	13 (6)	0 (0)	< 0.001	24 (2)
Cancer	112 (11)	32 (14)	44 (8)	34 (16)	2 (7)	0.004	6 (1)
Metastasis	24 (2)	8 (4)	10 (2)	5 (2)	1 (3)	0.514	27 (3)
Caregiver post-discharge, N (%)	563 (55)	140 (63)	292 (53)	121 (56)	10 (34)	0.066	150 (15)
Onset-to-door time, median minutes (IQR)	213 (80–672)	275 (72–959)	215 (93–641)	149 (63–384)	113 (41–368)	0.002	153 (15)

*IQR* Inter-quartile range, *SES* Socio-economic status, derived from status scores based on zip codes, *NIHSS* National Institute of Health Stroke Scale, *CCI* Charlson comorbidity index, *TIA* Transient ischemic attack, *PAOD* Peripheral arterial occlusive disease

The 3-month mortality was 24.5% (Table 2). The unadjusted difference in 3-month mortality rates between the four stroke centers was significant (*p* < 0.001). 581 (57%) of all patients had a favorable degree of disability (mRS < 3). There was also a significant unadjusted difference in mRS scale scores between the four stroke centers. The median EQ-5D index score at 3 months for all patients was 0.65 (inter-quartile range 0.10–0.83), and the unadjusted difference across the four stroke centers was also significant (*p* < 0.001). Missing mRS outcomes were 1205/2733 (44.1%) in the original database, with most missing mRS data being observed in stroke center IV (192/238 = 80.7%) (data not shown).

**Table 2:** Outcome Measures of Ischemic Stroke Patients (*N* = 1022)

Outcome variables	All patients (*N* = 1022)	Stroke Center I, University (*N* = 222)	Stroke Center II, District-based (*N* = 555)	Stroke Center III, District-based (*N* = 216)	Stroke Center IV, District-based (*N* = 29)	*P*-value
mRS at 3 months, N (%)						< 0.001
0	89 (8.7)	37 (16.7)	25 (4.5)	27 (12.5)	0	
1	262 (25.6)	62 (27.9)	143 (25.8)	53 (24.5)	4 (13.8)	
2	230 (22.5)	37 (16.7)	130 (23.4)	58 (26.9)	5 (17.2)	
3	108 (10.6)	23 (10.4)	67 (12.1)	15 (6.9)	3 (10.3)	
4	68 (6.7)	24 (10.8)	32 (5.8)	11 (5.1)	1 (3.4)	
5	15 (1.5)	2 (0.9)	11 (2.0)	2 (0.9)	0	
6	250 (24.5)	37 (16.7)	147 (26.5)	50 (23.1)	16 (55.2)	
EQ-5D index score at 3 months, median (IQR)	0.65 (0.1–0.83)	0.781 (0.45–1.00)	0.61 (0–0.78)	0.65 (0.28–0.83)	0 (0–0.60)	< 0.001

*mRS* Modified Rankin Scale scores, *EQ-5D* EuroQol 5-Dimension, *IQR* Inter-quartile range

### Case-mix models

Table 3 shows the remaining predictors in the regression models after backward selection for mortality, mRS and EQ-5D utility scores. The “strongest” (based on lowest *p*-values) independent variables in the model for mortality were age (OR = 1.07), NIHSS score on admission (OR = 1.17) and the Charlson’s comorbidity index (OR = 1.22). The strongest predictors for mRS at 3 months were age (OR = 1.04), NIHSS score at admission (OR = 1.17), heart failure (OR = 3.58) and previous stroke (OR = 1.74). There were only three overlapping predictors for the three different outcomes: age, NIHSS score on admission and heart failure. Exclusive predictors for the EQ-5D index score were sex (β = 0.041), socio-economic status (β = − 0.019), and nationality (β = − 0.074).

**Table 3:** Case-Mix Risk Adjustment Models for Mortality, mRS and EQ-5D

	Model 1*: Mortality at 3 months		Model 2*: mRS score at 3 months	Model 3*: EQ-5D index score at 3 months		
(Nagelkerke) R^2^	0.44		0.42		0.37	
AUC^a^	0.87		0.83		0.78	
Variables	Multivariable OR (95% CI)	*P*-value	Multivariable OR (95% CI)	*P*-value	ßeta (SE)	*P*-value
Age	1.07 (1.05–1.09)	< 0.001	1.04 (1.03–1.05)	< 0.001	−0.007 (0.001)	< 0.001
NIHSS on admission	1.17 (1.14–1.20)	< 0.001	1.17 (1.14–1.19)	< 0.001	− 0.020 (0.001)	< 0.001
Heart failure	2.54 (1.30–4.97)	0.007	3.58 (2.14–5.98)	< 0.001	− 0.130 (0.042)	0.002
Previous stroke			1.74 (1.33–2.27)	< 0.001	− 0.063 (0.023)	0.007
Smoking			1.58 (1.18–2.13)	0.003	− 0.055 (0.025)	0.028
Caregiver at discharge			0.67 (0.51–0.86)	0.002	0.057 (0.022)	0.010
Connective tissue disease			2.07 (0.93–4.61)	0.074	−0.119 (0.061)	0.053
Cancer			1.99 (1.35–2.94)	0.001	−0.065 (0.042)	0.122
CCI	1.22 (1.13–1.32)	< 0.001			−0.020 (0.007)	0.003
Onset-to-door time (per 10 min)	1.00 (1.00–1.00)	0.123				
Diabetes			2.09 (1.12–3.88)	0.020		
Sex (Ref: Female)					0.041 (0.020)	0.036
SES					−0.019 (0.010)	0.053
Nationality (Ref: Native Dutch)					−0.074 (0.045)	0.097

*SES* Socio-economic status, derived from neighborhood-based ranking, *NIHSS* National Institute of Health Stroke Scal, *CCI* Charlson comorbidity index, *R*^*2*^ R-squared, *95% CI* 95% confidence interval, *SE* Standard error

*The case-mix models was using backward selection with the Akaike information criterion (AIC) as a cut-off for the *p*-value. The imputed dataset (10 imputations of original data *N* = 1022) was used for the development of all three case-mix model development

^a^AUC values were calculated for mortality, dichotomized mRS scores (0–2 vs. 3–6) and dichotomized EQ-5D index scores (< 0.65 vs. ≥ 0.65)

The binary logistic regression model for mortality had an R^2^ = 0.44 (Table 3), compared to the ordinal regression model for the mRS which had an R^2^ = 0.42, and the linear regression model for the EQ-5D utility score with a R^2^ = 0.37. The largest increase in R^2^ was after the addition of NIHSS to the models for mortality and mRS, and age to the EQ-5D index score model (Fig. 3). After mRS and EQ-5D index scores were both transformed to a dichotomous outcome, AUC’s were compared between all three binary logistic regression models: AUC = 0.87 (mortality) vs. AUC = 0.83 (mRS ≥ 3) vs. AUC = 0.78 (EQ-5D index score ≥ 0. 65). As opposed to the models for mortality and mRS, it took more predictors in the model for EQ-5D index scores for the predictive ability to reach a plateau.

## Discussion

The objective of this study was to construct and compare case-mix adjustment models for three different outcomes, of which two were clinical (mortality and modified Rankin Scale at 3 months) and one patient-reported (EQ-5D utility score at 3 months). The three case-mix models had several predictors in common: age, NIHSS score at hospital admission, and heart failure. However, the most important difference in the case-mix adjustment models was that sex, nationality, and socio-economic status remained significant case-mix variables specifically for the PROM in contrast to the models for the clinical outcomes. It has to be stated that even if a predictor is significantly associated with the outcome, it doesn’t necessarily have to be included as a case-mix variable, if the prevalence distribution of the variable and its effect on the outcome of interest is similar across hospitals. The R-squared (R^2^) statistics of the model for the patient-reported outcome measure (PROM) was somewhat lower in comparison to the R^2^ statistics for mortality and the modified Rankin Scale (mRS), but contained more variables.

There have been multiple models previously developed and validated to predict clinical outcomes after stroke [11]. Bray et al. [26] developed and externally validated two case-mix models with 30-day post-stroke mortality as an outcome. The predictors included in the final models were similar to the findings of the current study: age, NIHSS on admission and atrial fibrillation. On the other hand, there has not been much research conducted on the development of case-mix factors for patient-reported outcomes (e.g. EQ-5D) in stroke care [27]. There was some overlap in the remaining case-mix variables in this study and those identified in previously published articles [28]. A review by Carod-Artal et al. [29] identified age, sex, stroke severity, physical impairment, functional status, and mental impairment as predictors for the health-related quality of life (HRQOL) after stroke. Mar et al. [30] also found the male gender and the NIHSS to be significantly associated with better EQ-5D values. The negative association between (history of) cancer and a lower quality of life (lower EQ-5D scores) in this study confirms previously published literature [23, 31, 32].

A striking observation is the caregiver presence post-discharge as a statistically significant predictor variable for mRS score at 3 months with an OR = 0.67 (95% CI 0.51–0.86; *p* = 0.002) and for the EQ-5D utility score at 3 months with a ßeta = 0.057 (SE 0.022). This observation of caregiver presence at hospital discharge being associated with a lower mRS score (better clinical outcome) and a higher EQ-5D utility score (better quality of life) at 3 months, highlights the potential benefits that a caregiver could offer (e.g. patient motivation, facilitating rehabilitative care) leading to improved functional status and quality of life. However, definite conclusions cannot be drawn about this association, because the definition of “caregiver” and “caregiver presence” was not similar across the multiple stroke centers; it was unclear if the absence of a caregiver implied no indication (e.g. low mRS score) or no need (admittance to a revalidation center or nursing home).

### Strengths and limitations

A considerable strength of this study is that it explores a relatively new field of research namely case-mix adjustments for PROMs in order to make inter-hospital performance comparisons. The case-mix variables for a PROM do not imply additional registration burden for recording data in quality registries because general (relevant) demographic variables (e.g. age, gender and socio-economic status) are already captured in standardized fashion. This is a major strength of the study.

An important limitation of this study is the notably large amount of missing outcome (mRS and EQ-5D) data in the original database. This problem is not uncommon in registries that are routinely acquired for the purpose of quality of care assessment, and it was the main reason this study solely focused on the development of case-mix risk adjustment models rather than benchmarking the included stroke centers. Although the estimated regression coefficients of all three case-mix models might be somewhat biased due to the substantial missingness, it is less important in this context and more about the differences in case-mix variables between the models. The missing data issue was partially countered by the use of multiple imputation for the predictor variables. The distribution of patient characteristics was compared between missing and non-missing mRS and EQ-5D groups (data not shown). These analyses showed significant differences in distribution in NIHSS score, SES rank (low, middle, high), nationality, and some cardiovascular comorbidities (hypertension, heart failure, hyperlipidemia) between missing and non-missing mRS and EQ-5D data. This observation implies that the generalizability of the final set of case-mix variables, observed in this study, should be corroborated in future research.

Another limitation is the loss-to-follow-up bias in this stroke registry: if missing 3-month mRS and EQ-5D data could be attributed to either patients’ full recovery or an extended stay in a rehabilitation center/ nursery home, it is quite possible that known outcome data could be skewed (both directionalities possible), seeing it was mostly recorded at outpatient clinics. Other stroke registries (e.g. European Safe Implementation of Thrombolysis in Stroke-Monitoring Study (SITS-MOST) [33] and UK Sentinel Stroke National Audit Program (SSNAP) [34]) have also incorporated patient-reported outcomes, which are typically collected at 3–6 months post-discharge through diverse methods like face-to-face interviews, telephone interviews or mailed questionnaires [35]. As the collection of PROMs at these time points can be challenging due to varying post-discharge patient trajectories and/ or substantial resource requirements (personnel and costs), future research should focus on efficient methods to optimally capture PROM data as part of value-based stroke care. This is an essential step that should to be taken before (case-mix) risk adjustment models are further developed.

In this study, R^2^ values were compared to pseudo-R^2^ values although they are not directly comparable. However, the objective of this study was to showcase the differences in predictors between the three models. It has to be noted that some potentially relevant psychological case-mix variables (e.g. depression, anxiety, EQ-5D scores at baseline) were not recorded in the database, even though they could influence PROM responses and thus ultimately impact the case-mix adjustment model. This paper suggests that the specific predictors for the EQ-5D, based on this data, have not been found yet.

## Conclusions

In conclusion, this study shows that other predictors (e.g. psychological and social factors) should be considered as potential case-mix variables for patient-reported outcome measures (PROMs) than for clinical outcomes in ischemic stroke patients. It is important that these specific case-mix variables should be included in order to benchmark hospitals legitimately on PROMs. One of the principles of value-based healthcare is to benchmark clinical outcomes and PROMs across different diseases and healthcare providers/ institutions to ensure quality improvement and competition [36]. This study identified a low (er) socio-economic status to be specifically associated with lower EQ-5D index scores. Future research should focus on finding other important predictors specific to PROMs in acute ischemic stroke to be able to further develop valid case-mix models.

## Data Availability

The data that support the findings of this study are available from Hester Lingsma, PhD but restrictions apply to the availability of these data, which were used under license for the current study, and so are not publicly available. Data are however available from the authors upon reasonable request and with permission of Hester Lingsma, PhD.

## Abbreviations

EQ-5D: EuroQol-5 Dimension Questionnaire
mRS: Modified Rankin Scale
PROM: Patient-Reported Outcome Measure
